# Parameter optimization of PID controller for water and fertilizer control system based on partial attraction adaptive firefly algorithm

**DOI:** 10.1038/s41598-022-16425-7

**Published:** 2022-07-16

**Authors:** Mingqi Huang, Min Tian, Yang Liu, Yao Zhang, Jie Zhou

**Affiliations:** 1 College of Mechanical and Electrical Engineering, Shihezi University, Shihezi, 832000 China; 2 College of Information Science and Technology, Shihezi University, Shihezi, 832000 China; 3 University of the Cordilleras, 2600 Baguio City, Philippines

**Keywords:** Electrical and electronic engineering, Mechanical engineering

## Abstract

Proportional Integral Derivative (PID) control is the main control method in the process of agricultural water and fertilizer regulation, and its parameter setting directly affects the control effect of water and fertilizer regulation. However, the traditional PID parameters are adjusted manually such as using the critical proportionality method, which is time-consuming and difficult to achieve optimal control effects. To solve the optimal combination of PID control parameters and improve the control effect of water and fertilizer regulation, a partial attraction adaptive firefly algorithm (PAAFA) is proposed in this paper. Specifically, a partial attraction strategy is designed to speed up the convergence of the PAAFA and reduce the oscillation problem at the late stage of the algorithm. In addition, an adaptive inertia weight operator is proposed to balance the global search capability and local search capability of PAAFA and avoid the algorithm from trapping in the local optimum. Subsequently, to test the performance of PAAFA, the algorithm is subjected to a series of simulation experiments and bench tests with the latest methods, i.e., genetic algorithm (GA), Adaptive genetic algorithm (AGA), and firefly algorithm (FA) applied to PID parameter optimization problems. The simulation results demonstrate that the regulation times of the response curve of PAAFA-based PID control are reduced by 22.75%,10.10%and 20.61%, respectively, compared with GA, AGA, and FA. The bench test results show that the PAAFA-based PID control has the smallest relative error, and best control accuracy compared to GA, AGA, and FA, with an average relative error reduction of 3.99, 2.42, and 3.50 percentage points respectively.

## Introduction

Water and fertilizer integration technology integrates the irrigation process and fertilization process to realize water-saving and fertilizer saving in the agricultural process, which is one of the development directions of modern agriculture. Through fertilizer mixing tank, water pump, and drip irrigation pipe network, the irrigation and fertilizer application system adds water-soluble fertilizer to irrigation water and delivers it to the roots of crops to achieve the purpose of water supply and fertilizer on-demand and water-saving irrigation^1,2^. In the process of irrigation and fertilization, the irrigation and fertilization device precisely controls the water supply and fertilization amount within the optimal control range to facilitate the development of the crop root system and crop growth^3^. In addition, the uniformity and stability of water and fertilizer flow in irrigation and fertilization system are related to the control precision of crop fertilizer amount. Therefore, precise control of water and fertilizer regulation according to crop water and fertilizer requirements is the key to realizing water-saving irrigation.

Since the water and fertilizer regulation process of irrigation and fertilizer system has problems of nonlinearity, time-varying, and hysteresis, which can affect the accuracy and stability of water and fertilizer control irrigation and fertilizer system, a control method with high control accuracy and good stability is needed. Because traditional PID controller has the advantages of a simple algorithm, good robust stability, high reliability, low cost, and a wide range of applications, it has become one of the main methods in irrigation and fertilization process control^4–7^. At present, users can achieve the required control accuracy and stability by adjusting the corresponding parameters of the PID controller of the irrigation and fertilization system, to realize the integrated irrigation and fertilization of crops and achieve better control results.

The control effect and stability of PID control mainly depend on the structure of the PID controller and the combination of three control parameters KP, KI, and KD. Therefore, improving the structure of PID controller and solving the optimal combination of PID control parameters are two main research directions to improve the control effect of PID and the parameter tuning of PID control is the best combinatorial optimization problem in NP-hard problems^8–10^. The traditional PID parameter adjustment methods, such as the decay curve method and the Ziegler-Nichols step response method, are mostly performed by manual experience, which make the parameter adjustment process of PID control complicated and tedious^11^. In addition, the traditional PID parameter tuning method can’t produce the best combination of the three control parameters KP, KI, and KD, which is unable to fulfill the control demands of the irrigation and fertilization system and is difficult to adapt to the needs of modern agricultural automation^12^. Therefore, how effectively realizing the parameter optimization of PID control becomes the key to improving the PID control technology.

In recent years, inspired by biology, the academic community proposed to use swarm intelligence algorithms for PID control parameter optimization, such as ant colony algorithm (ASO), GA, etc^13–15^. However, in the process of optimizing PID control parameters, the swarm intelligence optimization algorithms have some problems, such as complex parameter setting, limited global optimization capability, weak adaptability, and low precision. The FA is a novel swarm intelligence algorithm, which has been widely used in scientific computing and engineering applications due to its simple algorithm idea, few parameters to be adjusted, and easy implementation of the program^16,17^. Specifically, FA shows better performance in many scientific problems, but it still has some limitations, such as slow convergence and the tendency to trap local optimality in complex problems.

Therefore, this paper proposes a novel partial attraction adaptive firefly algorithm (PAAFA) to perform parameter optimization of PID control. Firstly, a partial attraction strategy for firefly individual renewal is proposed to speed up the convergence of the algorithm and reduce the oscillation problem. In addition, an adaptive inertia weight operator is designed to avoid the algorithm from trapping in the local optimum at a later stage. Subsequently, through a series of simulation experiments, this paper proves that PAAFA can effectively optimize PID control parameters and improve the control effect and stability of PID control.

The main purpose of this paper is to improve the PID control effect of the water and fertilizer regulation process by solving the optimal parameter combination of PID control through PAAFA under offline conditions. The major achievement of this paper is listed as follows:

1. A mathematical model of a flow control system in the water and fertilizer regulation process is established and a novel PAAFA is proposed. The PAAFA combines the advantages of partial attraction strategy and adaptive operator and is applied to the water and fertilizer regulation process for PID parameter optimization of irrigation and fertilizer application system, which greatly improves the control accuracy of PID control.
2. A new partial attraction strategy for individual renewal of fireflies is proposed. The attraction strategy can reduce computational time complexity and speed up the convergence of the algorithm while maintaining the population diversity. In addition, it can reduce the number of firefly movements and the oscillation problem of the PAAFA at the late stage of the algorithm.
3. A new adaptive inertia weight operator is proposed. The operator dynamically changes the weights of the position update formula according to the number of iterations, so it can equilibrate the global search capability and local search capability of the algorithm and avoid trapping in the local optimum.

The organization of this paper could be formulated as follows. In Section “Related work”, the research work related to the optimization of PID control parameters in water and fertilizer integration systems is presented. In Section “Mathematical model of PID control system for the water and fertilizer integration system”, the PID control system mathematical model of the water and fertilizer integration system is established. In Section “PID parameter optimization based on PAAFA”, PAAFA is proposed for parameter optimization of PID controllers. In Section “Results and discussion”, the simulation outcome and discussion about the algorithm performance of PAAFA are presented. Finally, the conclusion section is given in Section “Conclusion and future outlook”.

## Related work

The integration of water and fertilizer is a highly efficient and water-saving agricultural technology recognized in today's world. It mainly supplies water and fertilizer to crops accurately, regularly, and quantitatively at the same time by using irrigation equipment according to soil characteristics and crop growth rule^18^. Irrigation fertilization is a progressive fertilization technique that can replenish water and fertilizer to the crop at regular intervals, thus promoting the uptake of water and fertilizer by the crop^19^. Jing Hu et al.^20^ designed a comparative experiment to demonstrate that drip irrigation with integrated water and fertilizer technology improves water and nitrogen use efficiency and production stability compared to conventional diffuse irrigation and over-fertilization. Therefore, precise control of water and fertilizer regulation process using reasonable water and fertilizer saving technologies in agriculture is an important tool to achieve sustainable agricultural development.

PID control is the most popular and simple closed-loop controller for the water and fertilizer regulation process, which can achieve the required control accuracy and stability, and control effect by adjusting the corresponding parameters. Yubin Zhang et al.^21^ designed a control technique based on PID control for precise control of water and fertilizer density in agricultural fertilization and irrigation period, and the results showed that this PID control system has the advantage of high control accuracy. However, the control performance of this control system decreases when the fertilizer density varies greatly. To ensure the control accuracy, Boyu Wang et al.^22^ used PID to control the water and fertilizer ratios and established an online parameter setting model using RBF neural network to achieve accurate and fast ratio control. The results showed that the control effect of RBF-PID is more precise and steady than PID control. Teresa Arauz et al.^23^ designed a PI controller based on linear matrix inequality (LMI) to solve the optimal control issue. Simulation results presented that the novel controller can improve the control effect by 30% and can effectively control the irrigation canal water level. Due to the problems of nonlinearity, time-varying, and hysteresis in the water and fertilizer regulation process, which will affect the accuracy and stability of water and fertilizer control, the accuracy of the above conventional PID control still does not fulfill the expected demands.

In the control process, PID parameter setting often uses an empirical trial method to gradually adjust the proportion, integral and differential coefficients to achieve the desired control effect, such as the relay feedback PID parameter adjustment method^24^. These methods need to rely on experience and repeated debugging to rectify the PID parameters with time-consuming and labor-intensive work. In addition, the control precision can’t meet the requirement when the traditional PID Parameter tuning method is used in modern water and fertilizer control. As science and technology continue to develop, the control object in the actual engineering field shows characteristics such as time lag and nonlinearity, which makes it difficult for the traditional PID parameter adjustment method to achieve the optimal adjustment of PID parameters.

For complex PID parameter tuning problems, many scholars use swarm intelligence algorithms to optimize PID controller parameters. Zhang et al.^25^ designed a control model combining PID control, fuzzy control, and gray predictive control for water-fertilizer ratio adjustment and irrigation control accuracy in agricultural water-fertilizer irrigation. Hekimoglu et al.^26^ proposed the atomic Search Optimization (ASO) algorithm and its modified version to determine the control parameters of the PID controller for motor speed. To enhance the control performance of gas turbines, a hybrid control technique based on a modified particle swarm optimization algorithm (PSO) and cuckoo search algorithm (HIPSO_CS) is proposed by Yang et al.^27^ for PID parameter adjustment. The simulation outcomes presented that the gas turbine controlled by the fuzzy PID controller based on HIPSO_CS has a fast system response and good control stability. However, conventional swarm intelligence optimization algorithms, such as ACO, GA, etc., have issues such as complex parameter settings, the high computational complexity of algorithms, and limited global optimization capability.

The FA has the advantages of a clear evolutionary mechanism, fewer parameter settings, and better low-dimensional search capability, so it has become one of the important algorithms in the field of evolutionary computation recently. Jagatheesan et al.^28^ compared the performance of the proposed FFA-PID algorithm with that of GA (GAPID) and PSO (PSOPID) based PID controllers for the same power system, and the results showed that the FFA-based PID control system has the shortest steady-state time. You et al.^29^ proposed a method to optimize the PID control parameters by applying an improved FA with an adaptive step operator, and the simulation results presented that the method improves the control accuracy of the system, which led to better control of the AUV motion. Wang et al.^30^ proposed a neighbor-attraction-based firefly algorithm (NaFA), which first places all fireflies on a ring topology, and then takes k fireflies each in front and behind as neighbors of firefly I to guide its movement, reducing the occurrence of oscillation phenomena and enhancing stability. Yu^31^ introduced a probability parameter P to control the attraction frequency of the firefly, and this method is called the partial attraction model. However, the algorithms such as GA, FFA, and NaFA proposed by the above researchers applied to PID parameter optimization are slow to converge and prone to trap in local optimum in complex problems.

To optimize the parameters of PID control and improve the control effect of PID control, a novel PAAFA is proposed in this paper to perform parameter optimization of PID control. A partial attraction strategy is proposed to minimize the time complexity of the algorithm and the oscillation problem in the late convergence of the algorithm. In addition, an adaptive inertia weight operator is designed to avoid the PAAFA from trapping in the local optimum at the late stage.

## Mathematical model of PID control system for the water and fertilizer integration system

### Problem description

**Figure 1:**
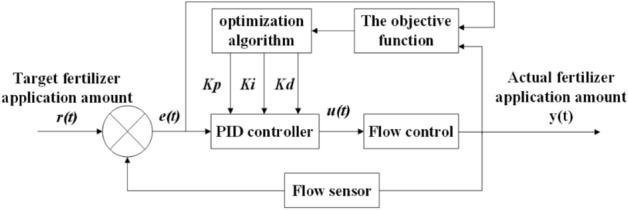
Schematic diagram of the process of optimizing PID parameters.

To optimize the PID control parameters of the water and fertilizer regulation process and reduce the response time of the control system of irrigation and fertilizer device, a flow control mathematical model of the water and fertilizer regulation process is established in this paper. In the process of water and fertilizer regulation, the control system of the irrigation fertilizer device mainly completes the quantitative control of fertilizer flow, and its control structure block diagram is shown in the Fig. 1. In this process, the control system takes the target fertilizer application amount $r(t)$ given by the irrigation fertilizer device as the input. The flow sensor collects the actual fertilizer application amount $y(t)$ and transmits it to the control system. The control system calculates the deviation $e(t)$ between the target fertilizer application amount $r(t)$ and the actual fertilizer application amount $y(t)$ and passes it to the PID controller. Then, the PID controller computes and gives the export $u(t)$. According to the $u(t)$, irrigation and fertilization device controls the flow rate of fertilizer in the pipeline through the inverter and asynchronous motor, and finally achieves the accurate control of fertilizer flow.

### Mathematical model of flow control system

Since the water and fertilizer regulation process of an irrigation and fertilizer application system is a time-lagged, nonlinear control object, it is hard to obtain an exact mathematical model, and related studies typically approximate the flow control model of the water and fertilizer regulation process as an equivalent.

As the fertilizer outlet pipe of the irrigation and fertilization system is filled with fertilizer, the velocity of fertilizer in the pipe will gradually rise and reach a stable state, which can be considered a first-order inertia link. Therefore, the mathematical model of fertilizer outlet pipe can be approximately equivalent to a pure lag first-order inertia link, which can be expressed as formula (1):

1 $$ {\text{G}}_{1} {\text{(s) = }}\frac{{k_{1} }}{{T_{1} s + 1}}e^{ - \tau s} $$

where $T_{1}$ is the inertia time constant of the fertilizer outlet pipe; $k_{1}$ is the gain of the fertilizer outlet pipe; and $\tau$ is the time lag constant of the fertilizer outlet pipe.

In this paper, ignoring the electromagnetic inertia of the three-phase asynchronous motor, and then doing a series of simplifications, approximations, and using linearization near its static operating point, the transfer function of the three-phase asynchronous motor after linearization can be derived as formula (2):

2 $$ {\text{G}}_{2} (s) = \frac{{k_{2} }}{{T_{2} s + 1}} $$

where $T_{2}$ is the time constant of inertia of the motor; $k_{2}$ is the gain of the motor.

The flow control system of the irrigation and fertilization device realizes the soft start process of the three-phase asynchronous motor through the frequency converter. Typically, the inverter is set to ramp feed, i.e., an integration link with a settable integration time is added to the frequency setting side. In this process, the inverter can be approximated as a proportional link because the time parameter of the inverter is much smaller than its hysteresis time parameter. In addition, because relay control and flow detection can also be regarded as proportional links, the transfer function of the inverter and other links of the flow control system can be equated as formula (3):

3 $$ G_{3} (s) = k_{3} $$

where $k_{3}$ is the gain of the inverter and other links of the system.

In summary, the mathematical model of the flow control system can be regarded as composed of one first-order inertia link, one pure hysteresis of the first-order inertia link pure hysteresis link, and one proportional link in series, so the flow control system transfer function of the irrigation and fertilization device in this paper can be expressed as formula (4):

4 $$ G(s) = G_{1} (s)G_{2} (s)G_{3} (s) = \frac{k}{{(T_{1} s + 1)(T_{2} s + 1)}}e^{ - \tau s} $$

where $k$ is the total system gain,$k = k_{1} \times k_{2} \times k_{3}$.

Because the transfer function of the flow control system depends on the structure and actual parameters of the system itself, the parameters of the transfer function $G(s)$ of the system can be determined after determining the structure and the hardware of the fertilizer pump and inverter, which are independent of other factors. Therefore, without considering external interference, the parameters $k,T_{1} ,T_{2} ,\tau$ of the flow control system expressed in formula (4) can be taken as 400, 1, 5, and 10 respectively, that is, the transfer function of the system can be formula (5):

5 $$ G(s) = \frac{400}{{(s + 1)(5s + 1)}}e^{ - 10s} $$

### The PID controller

The parameters of the PID controller, namely proportionality coefficient $K_{p}$, integration coefficient $K_{i}$, and differentiation coefficient $K_{d}$, have different impacts on the PID control effect. The proportional coefficient $K_{p}$ can adjust the deviation and improve the control sensitivity in a timely manner, but it cannot remove the steady-state error of the PID control system. The integral coefficient $K_{i}$ can remove the steady-state error of the system. The differential coefficient $K_{d}$ can the enhance response speed of the system and reduce the oscillation, but too large integral and differential coefficients will affect the stability of the system. Therefore, optimizing the parameters of the PID controller and rectifying its optimal combination can improve the control effect of the PID controller.

PID controller is a linear controller, which calculates the system deviation: $e(t) = r(t) - y(t)$ based on the system input $r(t)$ and the actual output $y(t)$. The PID controller processes proportional (P), integral (I), and differential (D) processing of the system deviation $e(t)$ and controls the controlled object by forming a linear combination. Its control law can be expressed as formula (6):

6 $$ u(t) = K_{p} [e(t) - \frac{1}{{T_{i} }}\int_{0}^{t} {e(t)dt + T_{d} \frac{de(t)}{{dt}}} ] = K_{p} e(t) + K_{i} \int_{0}^{t} {e(t)dt + K_{d} \frac{de(t)}{{dt}}} $$

Taking the Laplace transform of Eq. (6), the transfer function can be obtained as follows:

7 $$ G_{0} (s) = \frac{U(s)}{{E(s)}} = K_{p} + K_{i} \frac{1}{s} + K_{d} s $$

where $u(t)$ is the PID controller output; $T_{i}$ is the integration time constant; $T_{d}$ is the differential time constant; $K_{i} = \frac{{K_{p} }}{{T_{i} }}$ is the integration coefficient; $K_{d} = K_{p} *T_{d}$ is the differential coefficient.

## PID parameter optimization based on PAAFA

To solve the optimal combination of PID controller parameters, a PAAFA is proposed in this paper. FA has the characteristics of fewer parameter settings and strong low-dimensional search capability. Compared with other heuristic algorithms, FA has stronger local searchability, but it has the limitation of easily falling into local optimum. Therefore, in this paper, an adaptive inertia weight coefficient is added to PAAFA to effectively avoid the situation in which the algorithm falls into the local optimum. In addition, unlike the standard FA, this paper proposes a partial attraction strategy to substitute the attraction strategy of standard FA, which can reduce the algorithm time complexity and decrease the oscillation of the algorithm. Specifically, this paper solves the optimal combination of parameters of PID controller by using PAAFA, to reduce the overshoot and regulation time of PID controller response curve and improve the control effect of PID controller.

The implementation of PAAFA has the following three assumptions.

(i) Fireflies are gender-neutral, i.e., the mutual attraction between fireflies only takes individual luminance into account.
(ii) The attractiveness of fireflies is positively correlated with luminous luminance and negatively correlated with the distance between individuals.
(iii) The absolute luminance of the firefly depends on the objective function.

This section discusses the algorithmic process of PAAFA from algorithm coding and initialization, firefly luminance update, firefly attraction update, partial attraction strategy, firefly location adaptive update formula, and so on.

### PAAFA coding and initialization

In this paper, the parameters $K_{p}$,$K_{i}$ and $K_{d}$ of the PID controller are taken as the location parameters of fireflies in the three-dimensional space of PAAFA. Then, through PAAFA and related objective functions, the global optimal fireflies that meet the requirements can be solved.

The first step is to identify the encoding method of PAAFA. The PID controller parameters are real numbers, and the algorithm iteration of PAAFA is the update of the firefly spatial position, so the proposed algorithm of this paper adopts decimal encoding. For the PID controller parameter optimization problem, it is necessary to solve the best combination of,$$K_{p}$$$$K_{i}$$,$$K_{d}$$ 3 parameters, which corresponds to the spatial position of fireflies in three-dimensional space, so the decimal coding of individual fireflies can be expressed as formula (8):

8 $$ f_{i} = \left[ {x,y,z} \right] $$

In PID controller parameter optimization, the values of the three parameters have a certain range, so this paper takes 0 ≤ $x,y,z$ ≤ $U_{b}$; the initialization of the firefly population can be expressed as formula (9):

9 $$ Pop = rand(D,nPop)*Range $$

where $D = 3$ is the dimension of the solution space; $nPop$ is the size of the firefly population; $Range$ is the size of the range of values for the fireflies’ spatial position, $Range = U_{b}$.

After determining the coding of individual fireflies, the coding of the firefly population can be expressed as formula (10):

10 $$ Pop(i) = \left[ {\begin{array}{*{20}c} {x_{i} } & {y_{i} } & {z_{i} } \\ \end{array} } \right](i \in \{ 0,nPop\} ) $$

### Firefly luminance update

In the process of water and fertilizer regulation, the regulation time, overshoot, and error of the flow control system will affect the evaluation of the control effect, so the Integrated Time and Absolute Error (ITAE) is adopted to reflect the responsiveness and precision of the control system. ITAE is chosen as the objective function of PAAFA in this paper, and its formula is formula (11).

11 $$ J_{ITAE} = \int_{0}^{\infty } {t\left| {e(t)} \right|} dt $$

Because the control object of the flow control system requires a small change in the output of the PID controller, this paper corrects the formula (11) by adding the output control factor of the PID controller. According to the research of relevant scholars^32,33^, this paper sets the upper limit of the integral of Eq. (12) to be $t_{sim}$.In addition, according to the three assumptions of PAAFA, the luminance of the firefly is determined by the objective function of the PID parameter optimization algorithm, i.e., Eq. (12). Therefore, the corrected objective function formula can be expressed as formula (12).

12 $$ J_{NEW} = f_{i}^{t} = I_{i} = \int_{0}^{{t_{sim} }} {c_{1} t\left| {e(t)} \right| + c_{2} } u(t)dt $$

where, $c_{1}$, $c_{2}$ are the weight coefficients of ITAE and PID controller output respectively, $c_{1} + c_{2} = 1$;$t_{sim}$ is Simulation time;$I_{i}$ is the absolute luminance of the firefly $i$, i.e., the light intensity of the firefly $i$ at the light source ($r$ = 0).

Considering that the luminance of firefly $i$ decreases with increasing distance and the absorption of air, the relative luminance of firefly $i$ to firefly $j$ can be defined as:

13 $$ I_{ij} (r_{ij} ) = I_{i} e^{{ - \gamma r_{ij}^{2} }} $$

where $I_{ij} (r_{ij} )$ is the intensity of light from the firefly $i$ at the location of the firefly $j$, and the distance between the two is $r_{ij}$. $\gamma$ is the light absorption coefficient, which indicates the absorption rate of light by air, which affects the variation of the attraction $\beta_{ij} (r_{ij} )$, and is generally set as a constant. $r_{ij}$ is the Cartesian distance from the firefly $i$ to $j$,and its formula is:

14 $$ r_{ij} = \left\| {X_{i} - X_{j} } \right\| = \sqrt {\sum\limits_{k = 1}^{D} {(X_{i,k} - X_{j,k} )^{2} } } $$

where $X_{i}$,and $X_{j}$ are the space location of fireflies $i$, $j$, respectively; $k$ is the dimension of the spatial position.

### Firefly attraction update

In PAAFA, the size of firefly attraction determines its convergence speed and searchability of it. Assuming that the absolute luminance of the firefly $i$ is larger than that of the firefly $j$, the firefly $j$ is attracted to the firefly $i$ and moves toward the firefly $i$. The size of this attraction is dictated by the relative luminance of the firefly $i$ to the firefly $j$. The greater the relative luminance, the greater the attraction of the firefly. Therefore, the attraction $\beta_{ij} (r_{ij} )$ of firefly $i$ to firefly $j$ can be expressed as formula (15).

15 $$ \beta_{ij} (r_{ij} ) = \beta_{0} e^{{ - \gamma r_{ij}^{m} }} $$

where $m$ is usually taken as 2; $\beta_{0}$ is the initial attraction, i.e., the attraction at the source ($r = 0$), and $\beta_{0}$ can be taken as 1.

### Partial attraction strategy of PAAFA

**Figure 2:**
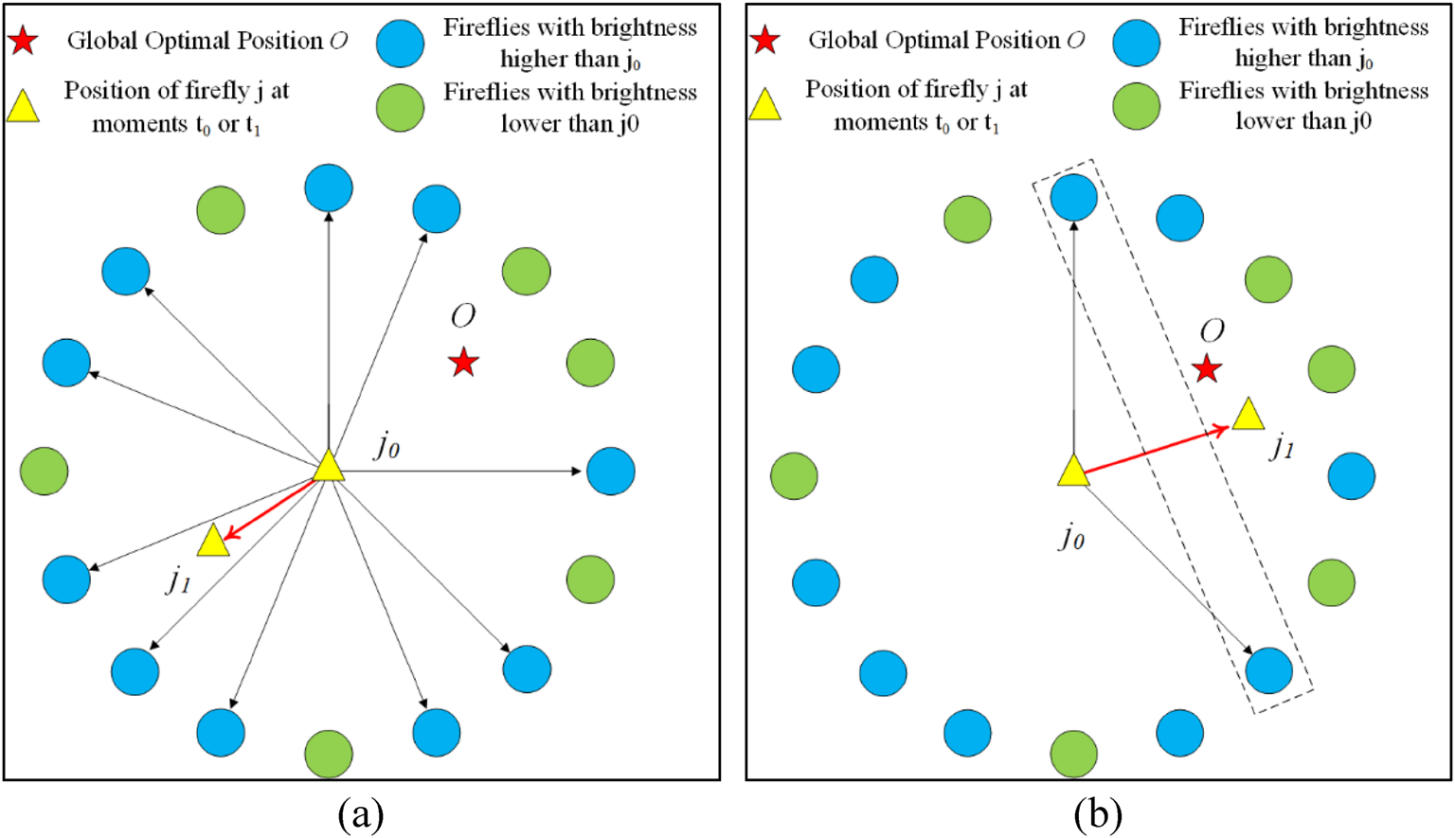
Schematic diagram of full-attraction strategy and partial-attraction strategy: (**a**) full-attraction strategy; (**b**) partial attraction strategy.

The control system of irrigation and fertilization system requires high control stability, so the parameter optimization algorithm of PID controller should have fast algorithm convergence speed and less possibility of algorithm oscillation. The standard FA firefly individual update uses the full-attraction strategy, whose strategy schematic is shown in Fig. 2a, i.e., each firefly is compared with other fireflies separately and moves once to each firefly that is brighter than it. The all-attraction strategy has two drawbacks, (i) the firefly is too much influenced by other fireflies during the movement, which causes too much oscillation during the movement and thus affects the convergence rate of the FA. (ii)When the population size $nPop$ is significant, each firefly has to be compared with other fireflies, so the computational time complexity of the algorithm will be higher.

To solve the problems of high computational complexity and slow convergence of FA, this paper proposes a partial attraction strategy for individual firefly updates, i.e., in the attraction strategy, each firefly will only be attracted to $m$ fireflies in the brighter firefly population and generate position updates. Specifically, firstly, all fireflies are sorted by luminance, the number of fireflies with higher luminance than the $i$-th firefly is determined as $U$ and the firefly population is selected as $UPop$.

Secondly, to reduce the computational time complexity of the algorithm and to maintain the population diversity in the algorithm, this paper introduces the Pareto principle^34,35^ (i.e. Key minority rule or the eighty-two rule) and roulette selection strategy to capture the main influencing factors in the population of the algorithm. In this paper, $m$ fireflies are selected from the firefly population $UPop$ to form an elite firefly population $mPop$, and the corresponding attractiveness and location updates are carried out. In this paper, the ratio of $m$ to $U$ is taken to be 0.2 according to the Pareto principle. Therefore, the number $m$ of the elite firefly population $mPop$ is calculated as formula (16).

16 $$ m = \left[ {0.2\;U} \right] + 1 $$

where, $1 \le U < nPop$; When $U = 0$, firefly $i$ is the brightest firefly $ibest$ of the current iteration. This firefly moves randomly, and the position update method is formula (18).

Figure 2 shows an example of a comparison between the full-attraction strategy and the partial-attraction strategy. Firefly $j_{0}$ is a firefly with luminance ranking 11, and the position of firefly $j_{1}$ is the position of firefly $j_{0}$ after the position update. In the full-attraction strategy, firefly $j_{0}$ is attracted to 10 brighter fireflies and moves 10 times to complete the position update, and its updated position is shown in Fig. 2a. Eventually, firefly $j_{0}$ moves 4 times toward the global optimal firefly ${\text{O}}$ and 6 times away from the global optimal firefly ${\text{O}}$. Therefore, the algorithm produces more oscillations. However, in the partial attraction strategy, the firefly $j_{0}$ is only attracted by 2 of the 10 brighter fireflies and moves 2 times each to complete the position update, and its updated position is shown in Fig. 2b. In this process, the firefly $j_{0}$ moves 2 times toward the global optimal firefly ${\text{O}}$ and does not move in the direction far from the global optimal firefly ${\text{O}}$. There is no oscillation in the algorithm. Therefore, the partial attraction strategy of PAAFA reduces the number of firefly movements, speeds up the convergence, and alleviates the oscillation phenomenon of the algorithm.

### Firefly location adaptive update formula

The parameters of the PID controller are expressed as the coordinates of the three-dimensional spatial position, and the firefly position update is directly related to the optimization of PID controller parameters. Attracted by Firefly $i$, Firefly $j$ shifts towards firefly $i$ and updates its position. The position update formula of firefly $j$ is shown in formula (17):

17 $$ X_{j} (t + 1) = X_{j} (t) + \beta_{ij} (r_{ij} )[X_{i} (t) - X_{j} (t)] + \alpha \varepsilon_{j} $$

where $X_{j} (t + 1)$ is the location of the firefly $j$ at a time $t + 1$; $X_{j} (t)$ is the location of the firefly $j$ at the time $t$. $\beta_{ij} (r_{ij} )[X_{i} (t) - X_{j} (t)]$ represents the displacement of firefly $j$ due to the attraction of firefly $i$. $\alpha \varepsilon_{j}$ is the perturbation term, where $\alpha$ is a random step, generally constant. $\varepsilon_{j}$ is a random number resulting from a uniform distribution, or some other distribution.

In addition, since other fireflies cannot attract the brightest firefly $ibest$ of the current number of iterations, the firefly $ibest$ moves its position randomly, and its position update formula is (18).

18 $$ X_{best} (t + 1) = X_{best} (t) + \alpha \varepsilon_{j} $$

In the late iteration of standard FA, the distance between fireflies becomes smaller and the attraction $\beta_{ij} (r_{ij} )$ becomes larger, which leads to an increase in the distance $X(t + 1)$ for updating the position of fireflies. Therefore, the combination of PID parameters oscillates repeatedly around the extreme value point in the late iteration of FA, which makes it impossible to solve for the optimal combination of PID control parameters.

To solve the above issues, an adaptive inertia weight coefficient formula and a firefly position adaptive update formula are proposed. The adaptive weight coefficient formula dynamically adjusts the size of the weight coefficient according to the algorithm iteration times and the current firefly adaptation value, which can avoid it from trapping in the local optimum. The adaptive inertia weight coefficient formula proposed in this paper is shown in formula (19).

19 $$ w(t) = \left\{ {\begin{array}{*{20}l} {(w_{\max } - w_{\min } )*\frac{{t_{\max } - t}}{{t_{\max } }} + w_{\min } } \hfill & {f_{i}^{t} \ge f_{avg}^{t - 1} } \hfill \\ {w_{\max } } \hfill & {f_{i}^{t} < f_{avg}^{t - 1} } \hfill \\ \end{array} } \right. $$

where $w_{\max }$, $w_{\min }$ are the maximum weight coefficients, minimum weight coefficients respectively, taken as $w_{\max } = 0.9$, $w_{\min } = 0.2$. $t$ is the current iteration number, $t_{\max }$ is the maximum iteration number. $f_{avg}^{t - 1}$ is the average objective function value of the $t - 1$ iteration, and its formula is shown in formula (20).

20 $$ f_{avg}^{t - 1} = \frac{{\sum\limits_{1}^{i} {f_{i}^{t - 1} } }}{nPop} $$

where $$i \in (1,nPop)$$.

The adaptive formula for firefly position update with the introduction of adaptive weighting coefficients can be equated as formula (21).

21 $$ X_{jNew} (t + 1) = w(t)X_{j} (t) + \beta_{ij} (r_{ij} )[X_{i} (t) - X_{j} (t)] + \alpha \varepsilon_{j} $$

### Termination conditions

If the loop of PAAFA meets the maximum number of iterations, the algorithm stops the loop and outputs the result, otherwise, the running step of the algorithm returns to step 4.2.

### Steps of PAAFA

*Step 1* The relevant parameters of PAAFA are initialized, and the fireflies in the population are randomly scattered in the solution space of the optimization problem.

*Step 2* The absolute luminance of fireflies are calculated by the location of fireflies and the objective function formula $J_{NEW} = f_{i}^{t} = I_{i} = \int_{0}^{{t_{sim} }} {c_{1} t\left| {e(t)} \right| + c_{2} } u(t)dt$. Fireflies with higher absolute luminance would attract fireflies with lower absolute luminance to move towards them.

*Step 3* Calculate the elite firefly population $mPop$ according to the partial attraction strategy.

*Step 4* Calculate the movement direction of the firefly with lower absolute luminance and its corresponding attraction size according to formula $\beta_{ij} (r_{ij} ) = \beta_{0} e^{{ - \gamma r_{ij}^{m} }}$ and the elite firefly population.

*Step 5* According to formula $X_{jNew} (t + 1) = w(t)X_{j} (t) + \beta_{ij} (r_{ij} )[X_{i} (t) - X_{j} (t)] + \alpha \varepsilon_{j}$, update the location information of fireflies with lower absolute luminance.

*Step 6* Using the firefly at the new location and the objective function formula $J_{NEW} = f_{i}^{t} = I_{i} = \int_{0}^{{t_{sim} }} {c_{1} t\left| {e(t)} \right| + c_{2} } u(t)dt$, update the absolute luminance of fireflies after location movement.

*Step 7* If the loop of PAAFA meets the maximum number of iterations, the algorithm stops the loop and outputs the result, otherwise, the running step of the algorithm returns to step 3.

**Figure 3:**
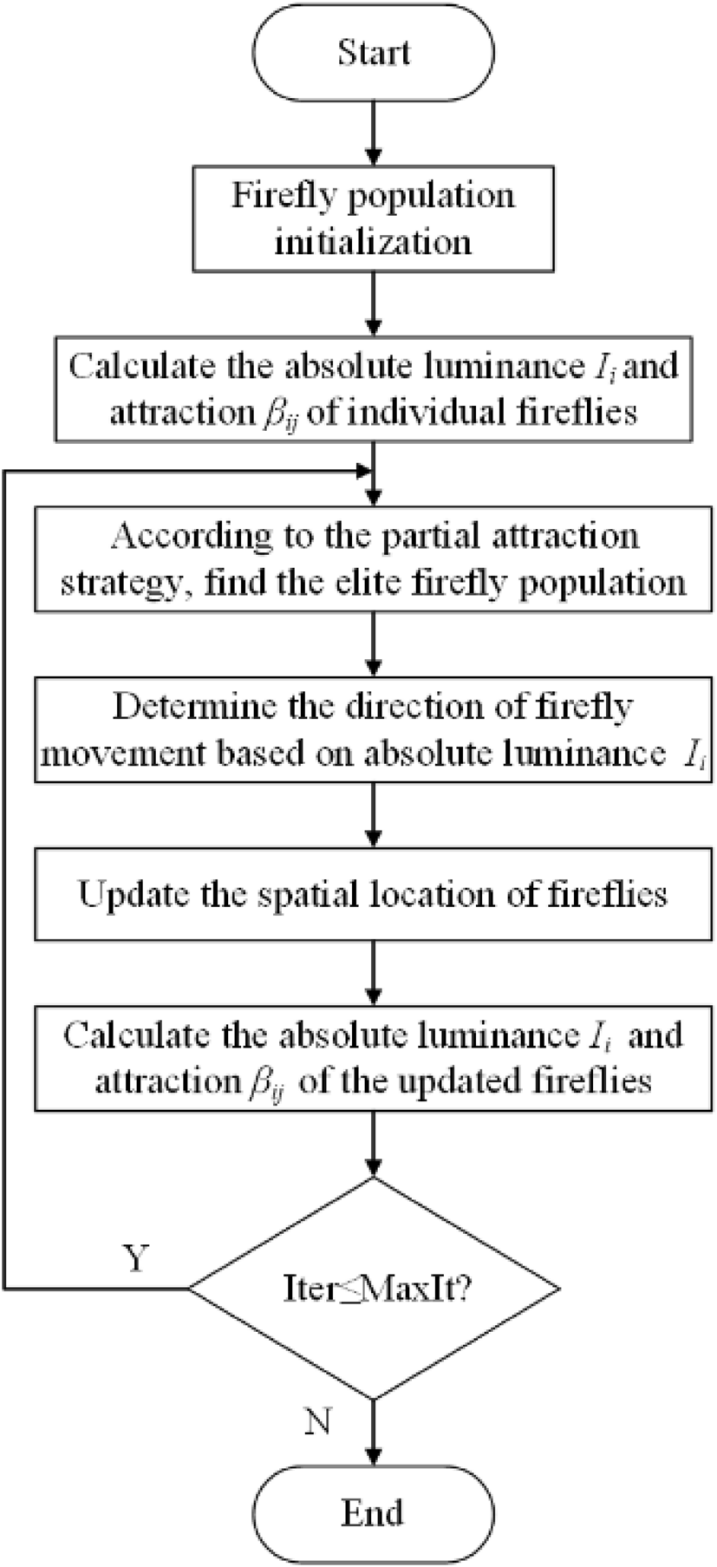
The flow chart of PAAFA.

The flow chart of PAAFA can be expressed in Fig. 3.

## Results and discussion

### Simulation results and discussion

To prove the capability of the proposed PAAFA in optimizing PID control parameters of the water fertilizer control system, a series of simulations are conducted and compared with GA, AGA^36^, and FA. The results of the simulation experiments, i.e., the comparison of optimal values and the unit step response curve of the PID, demonstrate the effectiveness of PAAFA. In addition to this, all the above experiments were performed using a Core i5 9th 3.00 GHz CPU machine and under other identical conditions, using Eq. (12) to calculate the optimal values of the PID parameters.

For the optimization problem of PID control parameters of the water fertilizer control system, the uniform definition of the common parameters helps to compare the algorithms in a relatively fair situation. Therefore, the largest number of iterations was set to 400 for all 4 algorithms. In addition, in PAAFA and FA, the light intensity absorption coefficient is 1, the initial attractiveness is 1, and the stochastic step size is 0.2. In PAAFA, the maximum adaptive inertia weight coefficient is 0.9 and the minimum adaptive inertia weight is 0.2. In GA and AGA, the crossover probability and variance probability of the population are 0.9 and 0.1, respectively.

#### Algorithm performance simulation experiment of PAAFA

**Figure 4:**
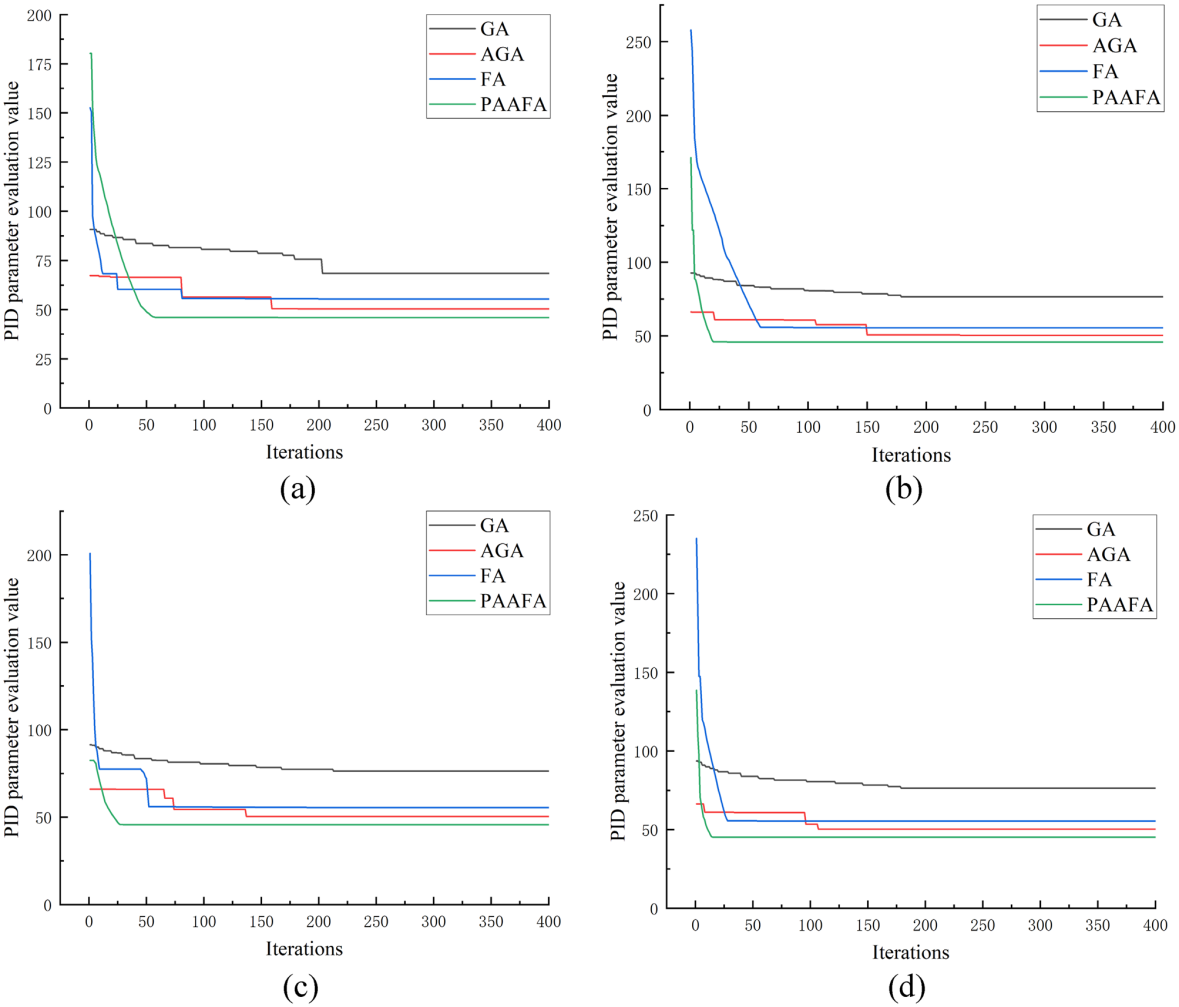
Comparison of PID parameter evaluation values for three algorithms: (**a**) population size of 30; (**b**) population size of 50; (**c**) population size of 70; (**d**) population size of 90.

Figure 4a–d show a comparison of PID parameter evaluation values for three algorithms. In general, the PID parameter evaluation values of PAAFA are better than those of GA, AGA, and FA for the population sizes of 30, 50, 70, and 90, respectively. Specifically, in Fig. 4a, the PID parameter evaluation values solved using the four algorithms are GA, FA, AGA, and PAAFA in descending order for the population size of 30, and the PID parameter evaluation value of PAAFA solution is the smallest. Therefore, Fig. 4a–d clearly show that PAAFA can use its good search capability to effectively avoid trapping in local optimum and achieve the search for the global optimum solution.

Table 1 presents the variation of the number of convergence iterations required for the four algorithms as the population size of the PID parameter optimization algorithm increases. At the population size of 30, the convergence iterations numbers required for the GA, AGA, and FA-based PID parameter optimization algorithm are 203, 159, and 81, respectively, while the PAAFA-based PID parameter optimization only requires 70 iterations, so the convergence speed of PAAFA is quicker than that of GA, AGA, and FA. When the population size increases to 50, 70, and 90, the convergence iterations numbers required to achieve convergence of PAAFA-based PID parameter optimization are 32, 38, and 14, respectively. From Table 1, it can be shown that the number of convergence iterations of PAAFA is fewer than that of GA, AGA, and FA, which can demonstrate that PAAFA has good convergence capability.

**Table 1:** Number of convergence iterations of the four algorithms for different population sizes.

Size of population	GA	AGA	FA	PAAFA
30	203	159	81	70
50	179	150	60	32
70	213	137	52	38
90	179	107	28	14

**Figure 5:**
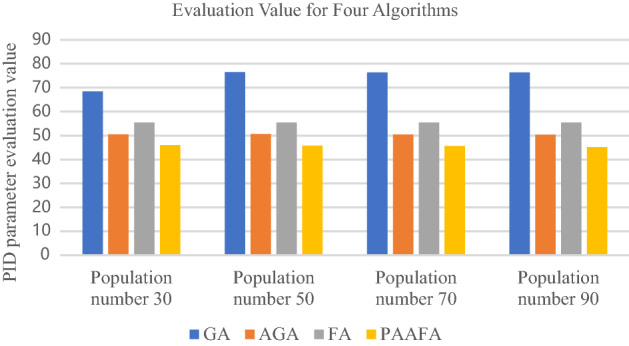
Comparison of PID parameter evaluation values of the four algorithms for different population sizes.

Figure 5 shows the PID parameter evaluation value of the four algorithms after solving the PID parameter. Specifically, the PID parameter evaluation value of PAAFA is optimal compared to GA, AGA, and FA, whether the population sizes are 30 or 50 or 70, or 90. In Table 2, the enhancements of the PID parameter evaluation value of PAAFA compared to GA, AGA, and FA are presented. In particularly, the PID parameter evaluation values of PAAFA are improved by 17.14%, 17.36%, 17.66%, and 18.46%, respectively, compared to FA. Therefore, the algorithm performance of PAAFA is the best among the four algorithms in solving the optimal combination of PID control parameters.

**Table 2:** Compared with the other 3 algorithms, the percentage of improvement in PID parameter evaluation value is optimized by PAAFA for different population sizes.

Size of population	GA (%)	AGA (%)	FA (%)
30	32.83	8.97	17.14
50	40.08	9.53	17.36
70	40.24	9.53	17.66
90	40.83	10.30	18.46

**Figure 6:**
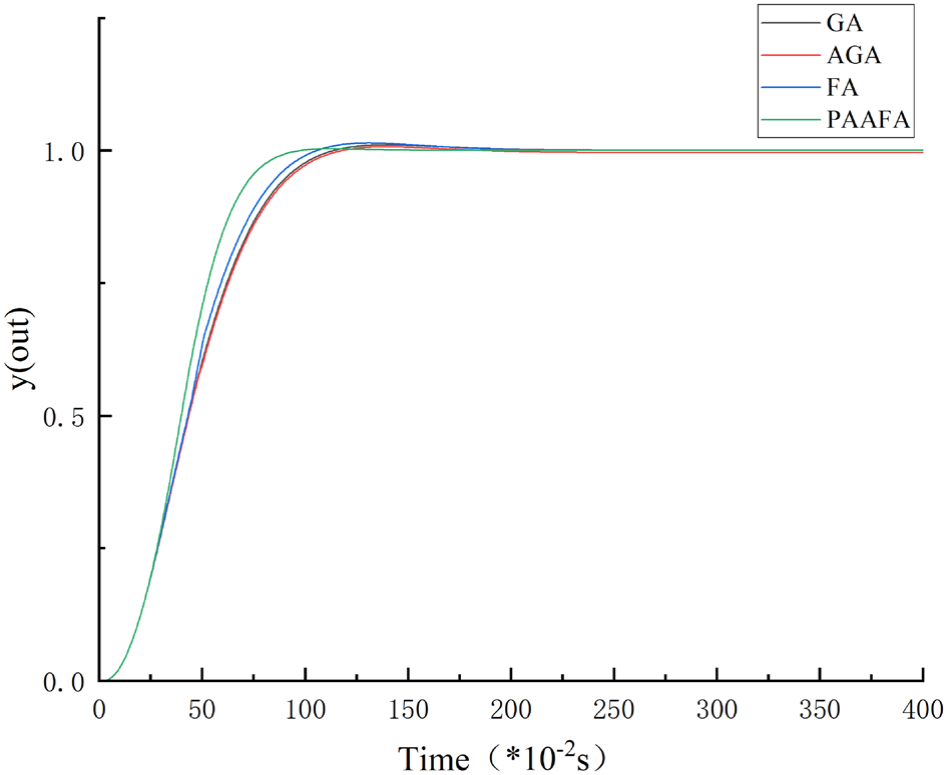
Unit step response curves of the 3 algorithms.

Figure 6 presents the unit step response of the four algorithms when the population size is 30. Specifically, for PAAFA-based PID control, the regulation time of the system is 2.58 s, the overshoot is 0.003, and there is a small disturbance after the system operation reaches stability. Compared with FA, the overshoot of PAAFA-based PID control is reduced by 0.011, the regulation time is reduced by 0.67 s, and the regulation time is 79.39% of it. Compared with GA, the overshoot of PAAFA-based PID control is reduced by 0.007, the regulation time is reduced by 0.76 s, and the regulation time is 77.25% of it. Compared with AGA, the overshoot of PAAFA-based PID control is reduced by 0.004, the regulation time is reduced by 0.29 s, and the regulation time is 89.90% of it. Overall, the PAAFA-based PID control has a more rapid system response, smaller overshoot, and better overall control effect.

#### Disturbance rejection performance test of PAAFA-based PID Control

**Figure 7:**
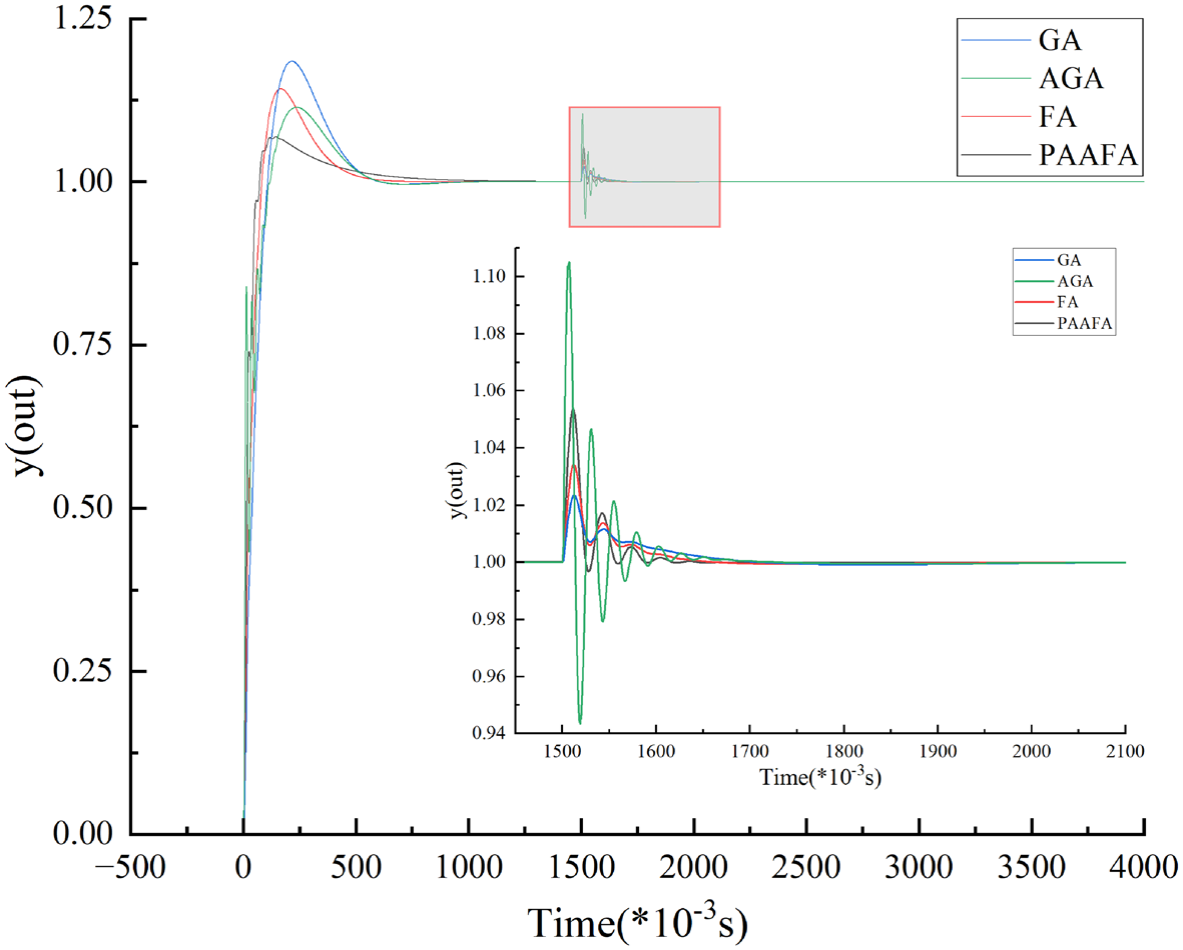
Unit step response under unit step disturbance.

To test the disturbance rejection performance of PAAFA-based PID control, one unit step disturbance was added to the system at 1.5 s. The results of the disturbance rejection performance test are shown in Fig. 7. The PID control based on four different PID parameter optimization algorithms all stabilizes the system output at the given value. In addition, after adding the unit step disturbance, the time to reach a steady state for GA, AGA, FA, and PAAFA-based PID control were 1.743 s, 1.764 s, 1.728 s, and 1.643 s, respectively. Compared with PAAFA-based PID control, the time to reach a steady state for GA, AGA, and FA-based PID control were increased by 6.09%, 7.36%, and 5.17%, respectively. Overall, the PAAFA-based PID control requires a shorter regulation time and has better disturbance rejection performance after adding unit step disturbance.

### Bench test results and discussion

#### Experimental materials and platform

**Figure 8:**
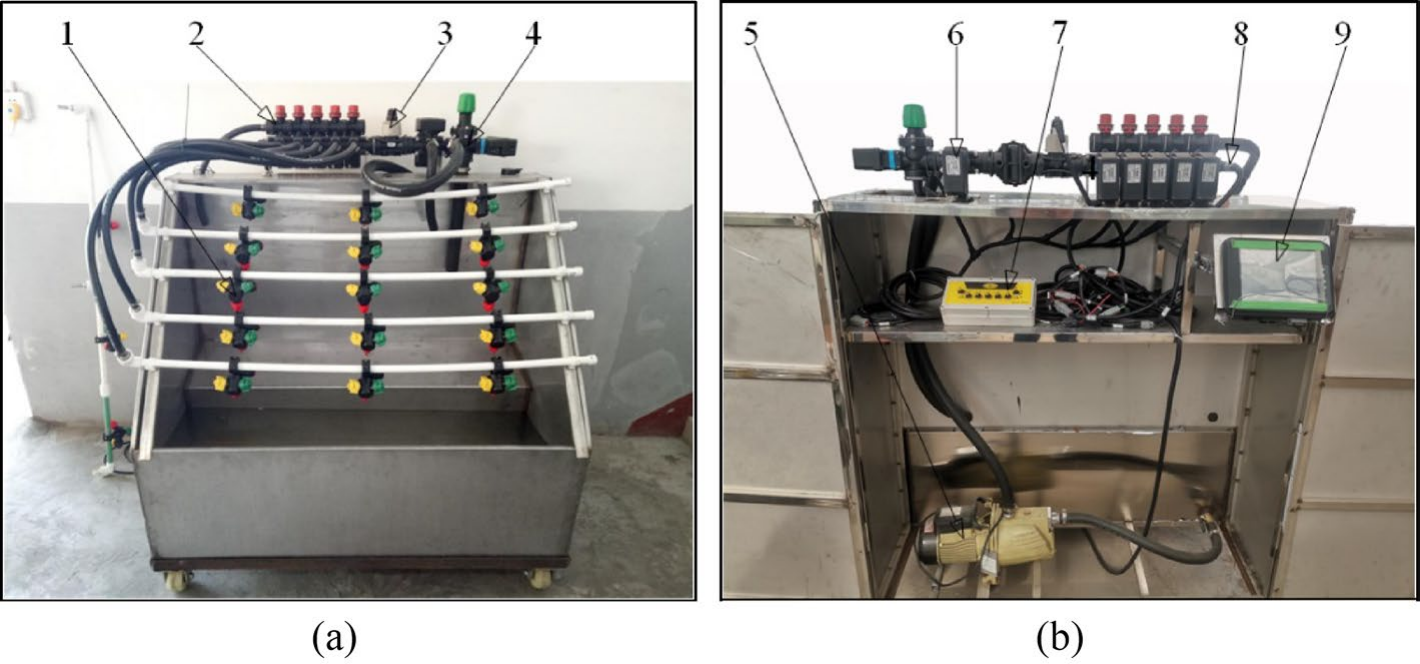
Bench test platform. 1. Spray nozzle; 2. Segmented valve group; 3. Flowmeter; 4. Electric main valve; 5. Self-priming jet pump; 6. Electric proportional valve; 7. Switch box; 8. Pressure sensor; 9. Controller. "Bench test platform" by Jinbin Bai is licensed under CC BY 4.0.

The flow control tests were carried out in a glass greenhouse at Shihezi University. The main installations of the bench test platform include control valve group ARAG 473, nozzle ARAG 422, filter ARAG326 9113, pipeline, ARAG WOLF flowmeter, electric proportional valve ARAG 463, self-priming jet pump JET 5-50-1.8, controller APC-3072, switch box, etc., as shown in Fig. 8. The height, length, and width of the test platform are 1.4 m, 1.5 m, and 0.6 m, respectively. Relevant parameters of the bench test platform are listed in Supplementary Table S1 online.

The control object of the test is an electric proportional valve, the test material is clear water without suspended solids. The accuracy of the fertilization flow control is measured and verified for GA-based PID control, AGA-based PID control, FA-based PID control, and PAAFA-based PID control respectively.

#### Discussion on bench test results

In this experiment, the control accuracy of the control system is reflected by the flow error. In this experiment, the absolute error of flow $\sigma_{a}$ represents the difference between the measured flow rate $Q_{m}$ of actual flow rate and the target flow rate $Q_{t}$; the relative error of flow $\sigma_{r}$ represents the ratio of the absolute error $\sigma_{a}$ to the target flow rate $Q_{t}$. The calculation formulas are shown in formulas (22) and (23).

22 $$ \sigma_{a} = Q_{m} - Q_{t} $$

23 $$ \sigma_{r} = \frac{{\sigma_{a} }}{{Q_{t} }} \times 100\% $$

where, $\sigma_{a}$ is the absolute error of the control system flow, $\sigma_{r}$ is the relative error of the system flow,%; $Q_{m}$ is the actual flow rate, L/min; $Q_{t}$ is the target flow rate read by the flow meter, L/min.

In this experiment, the flow meter reading on the controller screen is used as the agreed true value of the target flow rate (i.e. the target flow rate $Q_{t}$). In this experiment, four different target flow rates were selected for flow control experiments, namely 20, 30, 40, 50 L/min. For each different target flow rate, four sets of PID controller parameters are given by four PID parameter optimization algorithms. The flow output of the system under each set of PID controller parameters is measured five times, and the average value of the five measurement results is used as the measurement flow rate of the PID controller parameters of this group (i.e., the measurement flow $Q_{m}$ corresponding to the algorithm). According to the above measurement data, the absolute error and relative error of flow rate corresponding to each PID parameter optimization algorithm are calculated, and the experiment results are shown in Table 3.

**Table 3:** System flow control error.

Theoretical flow rate/(L min^−1^)	20	30	40	50
**GA-based PID**				
Measured flow rate/(L min^−1^)	21.13	31.49	42.31	52.41
Absolute error/(L min^−1^)	1.13	1.49	2.31	2.41
Relative error/%	5.65%	4.97%	5.78%	4.82%
**AGA-based PID**				
Measured flow rate/(L min^−1^)	20.73	31.17	41.43	51.91
Absolute error/(L min^−1^)	0.73	1.17	1.43	1.91
Relative error/%	3.65%	3.90%	3.58%	3.82%
**AGA-based PID**				
Measured flow rate/(L min^−1^)	20.99	31.41	41.94	52.37
Absolute error/(L min^−1^)	0.99	1.41	1.94	2.37
Relative error/%	4.95%	4.70%	4.85%	4.74%
**AGA-based PID**				
Measured flow rate/(L min^−1^)	19.75	30.41	40.59	50.58
Absolute error/(L min^−1^)	− 0.25	0.41	0.59	0.58
Relative error/%	− 1.25%	1.37%	1.48%	1.16%

As can be seen from Table 3, under the same test platform conditions and with different target flow rates, the relative error of the PAAFA-based PID control is lower than that of GA, AGA, and FA, and the control has the highest accuracy. The average relative errors of the GA, AGA, FA, and PAAFA-based PID controls were 5.30%, 3.74%, 4.81%, and 1.31% respectively, while the maximum absolute errors were 2.41, 1.91, 2.37, and 0.59 L/min respectively. The experiment results show that the PAAFA-based PID control has the lowest relative error, with an average relative error reduction of 3.99 percentage points compared to GA, 2.42 percentage points compared to AGA, and 3.50 percentage points compared to FA. Therefore, the PAAFA-based PID control has the best stability.

## Conclusion and future outlook

To optimize the PID controller parameters of an irrigation and fertilizer application system and to improve the control effect of its water and fertilizer regulation, a novel partial attraction adaptive firefly algorithm (PAAFA) is proposed. The major innovation of this paper is to propose the novel PAAFA and apply it to the optimization of PID controller parameters. Firstly, an adaptive inertia weight operator is designed, which effectively increases the search capability of PAAFA and avoids it from falling into the local optimum. Considering the rules of population updates, a partial attraction strategy is proposed to enhance the algorithm convergence rate and reduce the possibility of algorithm oscillations. Subsequently, the PAAFA is compared with the GA, AGA, and FA to demonstrate its effectiveness in optimizing the PID controller parameters. The simulation results indicate that the proposed PAAFA-based PID controller parameter optimization algorithm beats other algorithms in terms of algorithm convergence speed and jumping out of the local optimum. The PAAFA-based PID control system has improved the overshoot and regulation time in the system response curve and the disturbance rejection performance in the disturbance rejection test. The bench test results show that the PAAFA-based PID control has improved in both control accuracy and stability. Therefore, it can be concluded that the implementation of PAAFA can effectively improve the PID control effect of irrigation and fertilization devices.

Future research should consider the optimization of PID control parameters for more complex control systems, including but not limited to real-time online optimization of PID parameters, structural optimization of PID controllers, and the optimization of their corresponding parameters. In addition, in more complex cases, artificial neural networks in machine learning can be applied to PID parameter optimization research to further improve the control effect of PID control and enhance control stability.

## Supplementary Information

Supplementary Table S1.

## Data Availability

The datasets generated and analyzed during the current study are available from the corresponding author on reasonable request.
