# Supplementary material for: Parameter optimization of PID controller for water and fertilizer control system based on partial attraction adaptive firefly algorithm
Source: Sci Rep. 2022 Jul 16;12:12182. doi: 10.1038/s41598-022-16425-7 (PMC9288466; doi:10.1038/s41598-022-16425-7)
Supplement: Supplementary file 1 — Supplementary Table S1. [file 41598_2022_16425_MOESM1_ESM.docx]

**Supplementary Table S1.** Relevant parameters of the bench test platform.

| Serial number | Name of main device | Device type | Key parameters of the device |
| --- | --- | --- | --- |
| 1 | Self-priming jet pump | JET 5-50-1.8 | *Maximum Lift range*: 50 m |
|  |  |  | *Maximum suction range*: 9 m |
|  |  |  | *Maximum flow rate*: 83.3 L/min |
| 2 | The controller | APC-3072 | *Processor type*: Intel Atom TM E3845 |
|  |  |  | *Memory size*: 4G DDR3L |
|  |  |  | *Screen type*: five-wire resistive touch screen |
| 3 | Electric proportional valve | ARAG-463 | *maximum diameter of the electric proportional valve spool*: 19mm |
|  |  |  | *maximum flow rate under 0.15 MPa pressure*: 1016 L/min |
